# Online community engagement in response to COVID‐19 pandemic

**DOI:** 10.1111/hex.13194

**Published:** 2021-01-25

**Authors:** Logan Manikam, Shereen Allaham, Omar Zakieh, Yasmin Bou Karim, Isabel‐Cathérine Demel, Sayeeda Ali, Emma Wilson, Kate Oulton, Christopher Morris, Cally Tann, Hannah Kuper, Neha Batura, Clare Llewellyn, Andrew Hayward, Jenny Gilmour, Kelley Webb Martin, Carol Irish, Chanel Edwards, Monica Lakhanpaul, Michelle Heys

**Affiliations:** ^1^ Department of Epidemiology and Public Health University College London Institute of Epidemiology and Health Care London UK; ^2^ Aceso Global Health Consultants Limited London UK; ^3^ Department of Medicine Imperial College London London UK; ^4^ Department of Life Sciences & Medicine King’s College London GKT School of Medical Education London UK; ^5^ Population, Policy and Practice UCL Great Ormond Street Institute of Child Health London UK; ^6^ Great Ormond Street Hospital London UK; ^7^ University of Exeter Medical School University of Exeter Exeter UK; ^8^ Department of Infectious Disease Epidemiology London School of Hygiene & Tropical Medicine London UK; ^9^ International Centre for Evidence in Disability London School of Hygiene & Tropical Medicine London UK; ^10^ Institute for Global Health University College London London UK; ^11^ Department of Behavioural Sciences & Health UCL IEHC London UK; ^12^ Tower Hamlets GP Care Group Mile End Hospital London UK; ^13^ Children’s Health 0‐19 Service London Borough of Newham London UK; ^14^ Whittington Health NHS Trust London UK; ^15^ Specialist Children and Young People’s Services East London NHS Foundation Trust London UK

**Keywords:** children, community engagement, community‐based intervention, infant, online interventions

Community participatory research is a method of active engagement between community members to develop interventions that catalyse social change and improve health outcomes.^1^ Increasingly, particularly in low‐middle‐income countries (LMICs), participatory research is used to co‐develop low‐cost community interventions. UK health policy and providers are also placing increasing emphasis on strengthening community services to equitably optimize health outcomes. South‐to‐north translation of community‐based participatory interventions provides enormous potential to address these aims. In this article, we provide two examples of interventions which have been successfully implemented in LMICs^2^, ^3^, ^4^ and are currently undergoing adaptation and testing in the UK, driven by the emphasis of NHS policy shifting towards community strengthening.^5^ The first is community mobilization through participatory learning and action (PLA) cycles with women's groups and the second community‐based parent group programme for children with complex neurodisability (Ubuntu).^6^, ^7^ Here we describe how these programmes are being further adapted to run partially or fully online in response to COVID‐19, and lessons can be learnt from these two examples, which have accelerated digital innovation in the delivery of health services to enable non‐contact approaches.

Women's groups PLA cycle is a WHO‐recommended intervention to produce sustained improvements in maternal and newborn outcomes in rural LMICs. PLA cycles are iterative processes led by community facilitators (CFs) guiding participants through four‐stage cycles of identifying and prioritizing contextual health issues, designing strategies to address these issues and evaluating post‐implementation. The Nurture Early for Optimal Nutrition (*NEON*) programme^9^ aims to co‐adapt PLA from LMIC to theUK to improve infant feeding, care and dental hygiene practices for South Asian infants <2 years in East London.

Ubuntu family of programmes provides community‐based parent/carer programmes using participatory approaches. These programmes were developed in LMIC to support carers of children at risk or with a diagnosis of complex neurodisability. These are, for cerebral palsy: the Getting to Know Cerebral Palsy (*G2KCP*) programme, for young children with developmental disability: the ABAaNA Early Intervention Programme (*ABaANA: Uganda* */Rwanda*) and for children with congenital Zika syndrome (*Juntos: Brazil*).^10^ This approach is being adapted in the UK for families of children with complex neurodisability, such as cerebral palsy to address an expressed, unmet need by parents for such an intervention.^2^

Due to COVID‐19, the UK government enforced isolation measures, including social distancing.^11^ This affected community engagement studies that relied on face‐to‐face meetings such as *NEON* and community groups for complex neurodisability. Despite evidence of increasing health uptake and engagement, little is known about the feasibility of undertaking online community engagement and research, especially during a pandemic.^12^

Salmons (2016) introduced an e‐research framework that facilitates a holistic approach to undertake community engagement and qualitative research online.^13^ Furthermore, social media platforms have been found to be valid tools to engage with communities during times of crises.^13^, ^14^, ^15^ Consequently, the *NEON* team decided to adapt the intervention such that community engagement, delivery and evaluation occurred virtually, utilizing the software Zoom. CFs received a robust group and 1:1 training from the *NEON* team on how to use Zoom, to facilitate the *NEON* intervention meetings. Despite the varying digital literacy, all the CFs successfully used the online platform for the meetings. Similarly, the community parent/carer group intervention team have adapted their qualitative research methodology and workshops to online platforms.

Reflecting on our experiences, online engagement has several benefits over face‐to‐face; (a) CFs do not have to commute to meetings nor bear travel costs, and (b) participants have flexibility in terms of meeting dates and times. These factors helped in the conduct of meetings during holiday times and encouraged participation by reducing mobility barriers for physically disabled individuals and carers of infants. Meetings could also be recorded with the participants’ permission and accessed later to reflect on specific points and/or for those who missed meetings.

Since COVID‐19, there has been an increase in anxiety, depression and other mental health morbidity, which may have impacted on CF’s proactiveness to participate online. Participation promotion techniques, for example inviting participants by name to speak in turn, splitting the group into smaller discussion groups, were used to mitigate these issues. We recognize that community engagement is difficult during crises due to changing lifestyles and commitments with some CFs volunteering to deliver necessities in their community. Whilst not observed, participants may be reluctant to attend online meetings if they feel they lack privacy in their home setting. Whilst not always practically possible, we encouraged CFs to undertake meetings in a private room in their house. Other limitations and/or risks of virtual meetings include: (a) loss of body language and non‐verbal cues, particularly if individuals switch off their cameras and (b) risk worsening health inequities by reliance on digital methods as the highest risk, hardest to reach populations are likely those who experience greater digital poverty.^16^

After conducting online parent/carer workshops to gather feedback around the COVID‐19 experience, the *G2KCP* project team is undertaking qualitative focus group discussions (FGD) and semi‐structured interviews online. The team have incorporated solutions on how to conduct online qualitative research from Tuttas et al (2014) such as reducing FGDs sizes so that they are more manageable online.^17^ In addition, they are exploring the feasibility of digital implementation of the programme. Collaborators in Rwanda and Uganda are similarly currently exploring the digital adaptation of the ABaANA programme. To advance patient and public involvement and community engagement, an online Facebook group is being set up as a virtual support group for East London parents during the pandemic. Furthermore, this virtual community can act as a space to disseminate information and promote shared learning.

*NEON* and community parent groups for neurodisability represent two exemplar community participatory research projects adapting to COVID‐19 by implementing online research, meetings and intervention implementation as a successful alternative to face‐to‐face meetings. There may still be inevitable digital divide in the community, and evaluation of these is planned and required to ascertain impact on equity and efficacy. We hope that many generalizable lessons will be learned from our experiences that may be applied to the conduct of participatory and qualitative research and to the implementation of community‐based interventions in the UK and beyond.

## CONFLICT OF INTERESTS

No conflict of interests to declare.

## DATA SHARING AND AVAILABILITY STATEMENT

The data of this study are available from the corresponding author upon reasonable request.

## AUTHOR CONTRIBUTIONS

ML, LM, MH, NB, CL, AH and SA conceived the original concept of the study and designed the research methodology. SA carried out the ID meetings, workshops, co‐development of the intervention toolkit and wrote the paper. LM, ML, SA, NB, CL, AH, KO, CM, CT, HK, YBK, EW, JG, KWM, CI and CE validated the study and revised the manuscript critically for important intellectual content. OZ and ICD were involved in drafting materials for the NEON meetings and workshops of the Intervention Development Phase. OZ, ICD and SaA contributed to the manuscript writing, edited the final manuscript and prepared it for submission. LM and SA had primary responsibility for the final content. All authors read and contributed to reviewing the study data, the designing of the manuscript and the approval of the final manuscript.
